# Loss of YY1, a Regulator of Metabolism in Melanoma, Drives Melanoma Cell Invasiveness and Metastasis Formation

**DOI:** 10.3389/fcell.2022.916033

**Published:** 2022-05-26

**Authors:** Ulf Guendisch, Benjamin Loos, Phil F. Cheng, Reinhard Dummer, Mitchell P. Levesque, Sandra Varum, Lukas Sommer

**Affiliations:** ^1^ Institute of Anatomy, University of Zurich, Zurich, Switzerland; ^2^ Department of Dermatology, University Hospital Zurich, Zurich, Switzerland

**Keywords:** metabolism, melanoma, YY1, metastasis, EMT, cellular stress response, phenotype switching

## Abstract

Deregulation of cellular metabolism through metabolic rewiring and translational reprogramming are considered hallmark traits of tumor development and malignant progression. The transcription factor YY1 is a master regulator of metabolism that we have previously shown to orchestrate a metabolic program required for melanoma formation. In this study, we demonstrate that YY1, while being essential for primary melanoma formation, suppresses metastatic spreading. Its downregulation or loss resulted in the induction of an invasiveness gene program and sensitized melanoma cells for pro-invasive signaling molecules, such as TGF-β. In addition, NGFR, a key effector in melanoma invasion and phenotype switching, was among the most upregulated genes after YY1 knockdown. High levels of NGFR were also associated with other metabolic stress inducers, further indicating that YY1 knockdown mimics a metabolic stress program associated with an increased invasion potential in melanoma. Accordingly, while counteracting tumor growth, loss of YY1 strongly promoted melanoma cell invasiveness *in vitro* and metastasis formation in melanoma mouse models *in vivo*. Thus, our findings show that the metabolic regulator YY1 controls phenotype switching in melanoma.

## Introduction

For many years, it has been known that cancer cells adapt their metabolism to acquire necessary nutrients from a nutrient-poor tumor microenvironment to fulfill their altered energy expenditure (Pavlova and Thompson, 2016). Today, metabolic rewiring and translational reprogramming are considered important hallmarks of cancer not only for tumor establishment but also tumor progression and metastasis formation (Hanahan and Weinberg, 2011; Wei et al., 2020). In melanoma, the most aggressive type of skin cancer, metabolic and translational reprogramming has been postulated to be a conserved cellular stress response that can be induced by various tumor microenvironmental triggers, such as glutamine limitation, hypoxia or inflammation to drive invasion and determine therapeutic outcome (Falletta et al., 2017; Ratnikov et al., 2017; Wei et al., 2020). Besides, extrinsic triggers of metabolic reprogramming, cell-intrinsic oncogene-driven alterations can also influence metabolic rewiring in cancer (Min and Lee, 2018).

The ubiquitously expressed transcription factor Yin Yang 1 (YY1) plays a crucial role during development and tissue homeostasis regulating numerous cellular processes, such as proliferation, apoptosis, DNA repair and cell differentiation (Gordon et al., 2005). Several studies have identified a role for YY1 in diseases including cancer (Khachigian, 2018; Sarvagalla et al., 2019; Verheul et al., 2020). Similar to the diverging roles of YY1 during healthy tissue development, also in cancer YY1 has been found to act as either transcriptional activator or inhibitor and its overall impact on malignant tissues can be pro-as well as anti-tumorigenic (Sarvagalla et al., 2019). For instance, in pancreatic cancer, high YY1 expression levels are associated with better clinical outcome for patients (Chen et al., 2019), while in colon cancer YY1 was found to promote tumorigenesis (Fang et al., 2019). In melanoma, proper YY1 expression was found to be crucial for tumor initiation and formation in a transgenic melanoma mouse model (Varum et al., 2019). Thus, the cell type- and stage-dependent context of YY1 expression, i.e., the presence or absence of YY1 co-factors and interacting signaling pathways, seems to play a crucial role in the fine tuning of YY1’s function (Gordon et al., 2005; Zhang et al., 2011; Sarvagalla et al., 2019).

Recent studies have also implicated YY1 in regulating cell metabolism in cancer. In human hepatocarcinoma and cervical carcinoma, YY1 activated the rate-limiting enzyme in the pentose phosphate pathway hence promoting cancer cell proliferation (Wu et al., 2018). In melanoma, YY1 was identified to orchestrate metabolic pathways and protein synthesis crucial for cell survival and proliferation (Varum et al., 2019). Interestingly, the YY1-controlled transcriptional program observed in malignant melanoma cells resembled that of a YY1 program active in neural crest (NC) stem cells–the developmental origin of melanocytes and melanoma. In line with this, aberrant re-expression of markers reminiscent of NC stem cells in melanoma has been associated with tumor initiation, invasiveness and drug response (Restivo et al., 2017; Diener et al., 2021; Diener and Sommer, 2021).

However, while YY1 was found to be essential for primary melanoma establishment by direct regulation of metabolically relevant genes, it remains unclear what the functional outputs of these metabolic changes are with regard to tumor progression and metastatic spreading in already established melanoma.

## Materials and Methods

### Transgenic Melanoma Mouse Model

Lungs of *Tyr::N-Ras* ^*Q61K*^ *Cdnk2a* ^*−/−*^ *Tyr::Cre* ^*ERT2*^ *R26R::Stop:EGFP* *Yy1* ^*lox/lox*^ mice were isolated in the frame of the study of Varum et al. (2019), who also generated the transgenic mouse model.

In brief, *Tyr::N-Ras* ^*Q61K*^ *Cdnk2a* ^*−/−*^ *Tyr::Cre* ^*ERT2*^ *R26R::Stop:EGFP* *Yy1* ^*lox/lox*^ mice were obtained by crossing the *Tyr::NRas* ^*Q61K*^ and the *Cdnk2a* ^*−/−*^ mouse line (Serrano et al., 1996; Ackermann et al., 2005) with mice derived from Jackson laboratories that carried the transgenic alleles: *Tyr::Cre* ^*ERT2*^, *R26R::Stop:EGFP*, *Yy1* ^*lox/lox*^. Mice were crossed and bred in-house and mixed genders were used for this work. Genotyping was performed on toe clips. All animal breeding, housing and experimentation was conducted according to the guidelines of the veterinary office of the Canton of Zurich, Switzerland. More specifically, animals were housed in a controlled environment with a 12- h light/dark cycle, with free access to water and food.

To investigate the role of *Yy1* in metastasis formation, *Yy1* was conditionally depleted within the melanocytic lineage at 1 month of age by five consecutive intraperitoneal Tamoxifen (Cat#T5648, Sigma-Aldrich) injections (2 mg Tamoxifen diluted in ethanol and sunflower oil per injection). Animals were sacrificed at 5–6 months of age, when spontaneously developing tumors reached a tumor size of >2 mm or adverse clinical symptoms occurred including weight loss >15%, hunched back or any abnormal behavior. TM-injected *Tyr::N-Ras* ^*Q61K*^ *Cdnk2a* ^*−/−*^ *Tyr::Cre* ^*ERT2*^ *R26R::Stop:EGFP* *Yy1* ^*lox/lox*^ animals were used as experimental group and non-injected animals with the same genetic background were used as control group. To determine the number of lung metastases, lungs were dissected and immunohistochemically stained for the melanocytic marker DCT.

### Xenografts in Immunocompromised Mice

300, 000 human melanoma cells, lentivirally transduced with either an inducible shYY1 or shCtrl vector, were re-suspended in 100 µl of complete RPMI-1640 medium, mixed 1:1 with Matrigel matrix (356,234, BD Biosciences) and subcutaneously injected into the flanks of 10–14-week old female NUDE (Hsd:Athymic Nude-*Foxn1* ^*nu/nu*^; Envigo) using a 1 ml syringe and 25-gauge hypodermic needle. Tumors were grown to a maximum volume of 1 cm^3^, which was determined by tumor size measurements and the following formula: V = 2/3 × π × ((a+b)/4))^3^ with a representing tumor width and b representing tumor length, respectively.

For induction of the inducible YY1 knockdown *in vivo*, the drinking water of the mice was supplemented with 2 mg/ml doxycycline (D9891, Sigma-Aldrich) and 5% sucrose until the end point of the experiment was reached.

### Tissue Isolation of Lungs and Tumors From Xenografted Mice

At experimental end-point, tumors and lungs from immunocompromised mice were surgically removed, mechanically dissected into small pieces and digested using RPMI 1640 medium supplemented with 0.25 mg/ml Liberase DH Research Grade (05401054001, Roche). Following a 45 min incubation at 37°C, samples were treated with 0.2 mg/ml DNase I (10104159001, Roche) for 15 min at 37°C to generate a single cell suspension. Next, samples were filtered through 40 µm Falcon Cell Strainers (352,340, Thermo Fisher Scientific) to remove remaining tissue. Cell suspensions were washed with PBS, re-suspended in PBS containing 2 mM EDTA and RFP-positive cells were quantified by flow cytometry (lung tissue) or FACS sorted (tumor tissue). RFP-positive tumor cells were next submitted to RNA or protein isolation.

### Immunohistochemistry

Mouse lungs were fixed in ROTI®Histofix 4% (P087.1, Roth) overnight at 4°C and embedded in paraffin. The samples were processed into 5 µm thick sections and deparaffinization was performed as previously described (Zingg et al., 2015). Next, sections were subjected to heat-induced epitope retrieval in Citrate buffer (S2369, Agilent Dako) and rinsed with PBS. For blocking, in-house-blocking buffer (0.2% Triton X-100, 0.2% Bovine Serum Albumin, 0.2% Gelatin type A from porcine skin, 0.2% Casein, 0.001% Sodium azide, 1X TBS) was applied for 15 min. After washing with PBS, sections were stained with primary antibodies (Supplementary Table S1) diluted in Antibody Diluent (S0809, Agilent Dako) at 4°C overnight. The next day, samples were washed again with PBS and stained with secondary antibodies diluted in PBS (Supplementary Table S1) for 1 h at room temperature. Finally, samples were washed with PBS and mounted using Fluorescent Mounting Medium (S3023, Agilent Dako) containing Hoechst 33,342 (14,533, Sigma-Aldrich). Slides were imaged with the Mirax Slide Scanner (Zeiss).

### Protein Isolation and Western Blotting

For protein isolation, total cell lysates were prepared using RIPA buffer (89,900, Thermo Fisher Scientific) containing Halt Protease and Phosphatase Inhibitor Cocktail (78,440, Thermo Fisher Scientific). To determine protein concentrations, Bicinchoninic acid (BCA) Protein Assay (23,227, Thermo Fisher Scientific) was performed and absorbance measured at 562 nm wavelength using a BioTek Cytation five plate reader (Agilent). Protein samples were denatured for 5 min at 95°C with Laemmli Sample Buffer (1610747, Bio-Rad) containing 10% 2-mercaptoethanol. For SDS-PAGE, 20 µg of protein was loaded on a 4%–20% Mini-Protean TGX gel (456–1,094, Bio-Rad). Samples were transferred onto nitrocellulose membranes (1704158, BioRad) using a TransBlot turbo system (BioRad) and blocked for 1 h at room temperature (RT) with Odyssey blocking buffer (927–40,000, LI-COR Biosciences). Primary antibodies (Supplementary Table S1) were applied in Odyssey blocking buffer overnight at 4°C. Membranes were washed with PBS containing 0.1% TWEEN 20 (P1379, Sigma-Aldrich) before secondary antibodies (Supplementary Table S1) diluted in Odyssey blocking buffer were applied for 1 h at RT. Blots were scanned using the Odyssey imaging system (LI-COR Biosciences) and quantified in ImageJ.

### RNA Isolation and Quantitative Real-Time PCR

Total RNA was isolated using the RNAeasy mini kit (74,106, QIAGEN) following manufacturer’s instructions. Isolated and purified RNA was quantified using NanoDrop ND-2000 Spectrophotometer (Thermo Fisher Scientific). For cDNA synthesis, Maxima First Strand cDNA Synthesis kit (K1641, Thermo Fisher Scientific) was used according to manufacturer’s protocol. Quantitative real-time PCR (qPCR) was performed on a LightCycler 480 System (Roche) using LightCycler 480 SYBR Green I Master Mix (4707516001, Roche) and specific primers for the genes of interests (Supplementary Table S2). For quantification purposes, RNA expression was normalized to housekeeping gene transcripts indicated in the respective figures.

### siRNA Transfections

For transient YY1 knockdown, melanoma cells were transfected with Stealth siRNA YY1HSS111432 (Invitrogen) with the following sequence: CAU​CUU​AAC​ACA​UGC​UAA​GGC​CAA​A. As a control, scrambled siRNA (12,935, Thermo Fisher Scientific) was applied. JetPRIME (101000046, Polyplus) was used for all transfection experiments, following the manufacturer’s recommendations.

### Cell Lines and Cell Culture

The following cell lines have been used for this study: M010817 (established and genotyped by the URPP Live cell Biobank, University of Zurich), Skmel28 (HBT-72, ATCC), Mel888 (Rubinfeld et al., 1997) and HEK-293T (CRL-3216, ATCC). Human melanoma cells were grown in RPMI 1640 (42,401, Thermo Fisher Scientific) containing 10% fetal bovine serum (FBS, 16,140, Thermo Fisher Scientific), 4 mM L-Glutamine (25,030, Thermo Fisher Scientific) and Penicillin-Streptomycin (15,070, Thermo Fisher Scientific). HEK-293T cells were grown in DMEM/F12 (11320033, Thermo Fisher Scientific) supplemented with 10% FBS and Penicillin-Streptomycin. All cells were cultured in cell culture incubators (Binder) at 37°C with 5% CO_2_.

Metabolic stress experiments were performed as follows. For Glutamine starvation, cells were cultured in RPMI 1640 (42,401, Thermo Fisher Scientific) containing 10% fetal bovine serum (FBS, 16,140, Thermo Fisher Scientific) and Penicillin-Streptomycin (15,070, Thermo Fisher Scientific). For cycloheximide and oligomycin treatment, cells were grown in complete medium (RPMI 1640) and treated with 500 ng/ml of cycloheximide (C1988-5G, Merck) or 1 µM of oligomycin (103,010–100, Agilent), respectively. All treatments were performed for the time points indicated.

### Inducible shRNA Vector

Three doxycycline inducible shYY1 lentiviral vector plasmids were purchased from Cellecta together with a non-targeting shRNA in the same backbone, which was used as control. *In vitro*, YY1 knockdown was induced by supplementing media with 1 µg/ml of doxycycline for 48 h followed. *In vivo*, drinking water of the mice was supplemented with 2 mg/ml of doxycycline for the indicated time courses. Confirmation of YY1 knockdown was conducted by qRT-PCR and western blotting.

### Lentivirus Production and Transduction

For virus production, HEK-293T cells were transfected with the lentiviral packaging vector psPAX2 (12,260, Addgene), the lentiviral envelope vector pMD2.G (12,259, Addgene) as well as either the inducible shYY1 or shCtrl vector using the JetPRIME transfection kit (114–-15, Polyplus) according to manufacturer’s instructions. 48 and 72 h post-transfection, supernatants containing the lentiviral particles were collected and filtered through a 0.22 µm filter (83.1826, Sarstedt). Lentiviral transduction of melanoma cells was performed with the help of 8 µg/ml polybrene (H9268, Sigma-Aldrich). Melanoma cells were infected with lentiviral particle containing media for 24 h before media was replaced by complete growth media.

### Corning®Matrigel^®^ Invasion and Migration Assay

Migration and invasion assays were performed similar to the manufacturer’s guidelines. More specifically, for invasion assays, TranswellTM inserts (3,464, Corning) were coated with Corning^®^ Matrigel^®^ Matrix (354,234, Corning), which was diluted 1:35 with empty RPMI 1640 media (0% FBS, 4 mM L-Glutamine, Penicillin- Streptomycin). The coating was performed as follows. 50 µl of diluted Corning Matrigel Matrix was applied to each insert and incubated for 3 h at 37°C. Next, 200′000 human melanoma cells were resuspended in empty RPMI 1640 medium were transferred to each well on top of the Matrigel Matrix layer. Complete growth media, containing 10% FBS, was used as chemoattractant in the bottom wells of the assay. After 24 h, all cells that migrated/invaded through the membrane were collected and quantified using a Neubauer counting chamber. The migration assay was performed in a similar way except Transwell Inserts were not coated with Matrigel before cells were transferred to the assay.

### Bulk RNA-Sequencing Analysis

RNA sequencing of human melanoma cells was performed at the Functional Genomic Center Zurich, Switzerland. Total RNA of three experimental replicates per condition was isolated as described above. Quality control of the purified RNA was conducted on the Agilent RNA ScreenTape and Agilent 4,200 TapeStation. For poly-A mRNA enrichment, magnetic beads were used (TruSeq RNA Library Prep Kit v2) prior to cDNA synthesis and library preparation. RNA sequencing was performed with Illumina HiSeq4000. Quantification was achieved from single-end reads using STAR aligner and mapped to the *Homo sapiens* reference genome hg38. The bioinformatic analysis of differentially expressed (DE) genes was run with DeSeq2. All Gene Set Enrichment Analysis were performed with MetaCore™ (Thomson Reuters).

### Statistical Analysis

Information about number of animals used or n as well as statistical tests used are indicated and described in each figure. Statistics were carried out with GraphPad Prism6 and Excel. All experiment performed with cell lines were done at least in three independent biological replicates as well as with three technical replicates in each experiment. The error bars depicted in the graphs represent the mean ± SD. Significant differences between two groups were marked with asterisks (*p < 0.05; **p < 0.01; ***p < 0.001; ****p < 0.0001).

## Results

### YY1 Suppresses a Subset of Genes Involved in Invasion and Promotes Expression of Genes Involved in Proliferation and Differentiation

**FIGURE 1:**
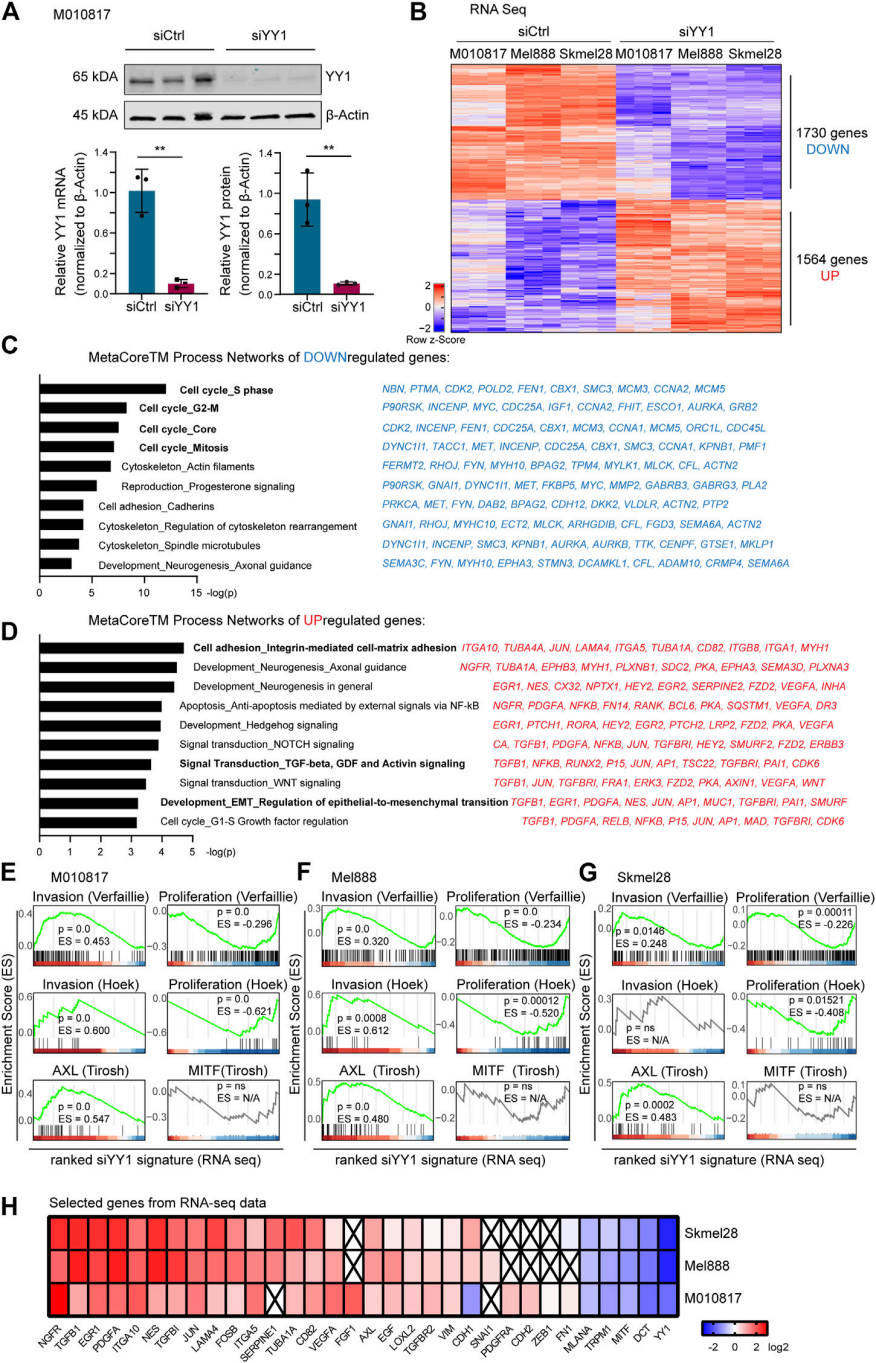
RNA sequencing of YY1 knockdown uncovers invasive gene signature. **(A)** YY1 expression after 48 h siRNA-mediated knockdown in M010817 cells. Top panel: western blot. Bottom panel left: Quantification of qRT-PCR. Bottom panel right: Quantification of western blot from top panel. All experiments have been performed in independent biological triplicates. Data is shown in fold change (FC) relative to control. *p* value was determined with unpaired Student’s t-test with *p*-values: *< 0.05; **<0.01; ***<0.001; ****<0.0001. Error bars represent mean ± SD. **(B)** Heatmap of differentially expressed genes of the human cell lines M010817, Mel888 and Skmel28 48 h after siRNA-mediated YY1 knockdown. 1730 genes were significantly downregulated, 1,564 genes were significantly upregulated. **(C)** Top 10 Metacore™ process networks of downregulated genes. Top 10 downregulated genes of each process are depicted next to them. **(D)** Top 10 Metacore™ process networks of upregulated genes. Top 10 upregulated genes of each process are depicted next to them. **(E–G)** Gene Set Enrichment Analysis (GSEA) of differentially expressed genes after YY1 knockdown (log2 ratio-ranked) with published gene signatures representing an “invasive,” a “proliferative,” an “AXL” and a “MITF” program. Cutoffs for B-G were set at Log2 ratio ≥0.58 or ≤ −0.58, *p* value < 0.05 and FDR <0.05. **(H)** Heat map representing genes selected from the RNA-seq data of three human melanoma cell lines.

To investigate the role of YY1 in human melanoma cells, we performed siRNA-mediated YY1 knockdown experiments in three human melanoma cell lines with different mutational backgrounds, namely M010817 (*NRAS* ^*Q61K*^-mutated), Mel888 (*BRAF* ^*V600E*^-mutated) and Skmel28 (*BRAF* ^*V600E*^-mutated). The molecular phenotype of all three human melanoma cell lines was determined as “proliferative” according to the previously published classification of melanoma cell lines by Widmer *et al.* (2012). After assessing the knockdown efficiency on mRNA and protein level (Figure 1A, Supplementary Figures S1A,B), we performed RNA-seq on siCtrl and siYY1-treated cells followed by differential gene expression analysis. 1730 genes were significantly downregulated and 1,564 genes significantly upregulated in siYY1-transfected cells compared to siCtrl cells (Figure 1B). Metacore™ process network analysis for downregulated genes indicated that YY1 positively regulates genes involved in cell cycle regulation (Figure 1C) in agreement with the previously reported role of YY1 in melanoma cell proliferation (Varum et al., 2019). Analysis of the upregulated genes revealed that YY1 negatively regulates genes involved in cell adhesion, TGF-β signal transduction and Epithelial-to-Mesenchymal transition (EMT) among others (Figure 1D). Thus, YY1 knockdown in human melanoma cells appeared to induce changes in gene expression reminiscent of a process referred to as “phenotype switching,” by which melanoma cells switch from a proliferative to an invasive state (Restivo et al., 2017; Rambow, Marine and Goding, 2019).

To verify whether there was a significant correlation of the siYY1-induced gene signature with published phenotype switching profiles, we performed gene set enrichment analysis (GSEA) of our RNA-seq data finding that it mostly positively correlated with invasiveness signatures and anti-correlated with proliferation signatures, respectively (Hoek et al., 2008; Verfaillie et al., 2015; Tirosh et al., 2016) (Figures 1E–G). Of note, among the differentially expressed genes most upregulated after YY1 knockdown were NGFR and TGFB1, two well-known inducers of EMT and EMT-like phenotype switching (Xu, Lamouille and Derynck, 2009; Restivo et al., 2017). On the opposite, we observed a strong downregulation of the melanocyte differentiation marker MITF and its downstream targets, DCT, TRPM1 and MLANA (Figure 1H). MITF has also been reported to be involved in melanoma phenotype switching, in that low levels of MITF promote a more invasive/less proliferative melanoma cell phenotype (Goding, 2011). Together, these findings suggest that YY1 is involved in melanoma invasion and phenotype switching.

### Metabolic Stress Associated With Loss of YY1 in Human Melanoma Cells Induces Expression Changes in Phenotype Switching Genes

**FIGURE 2:**
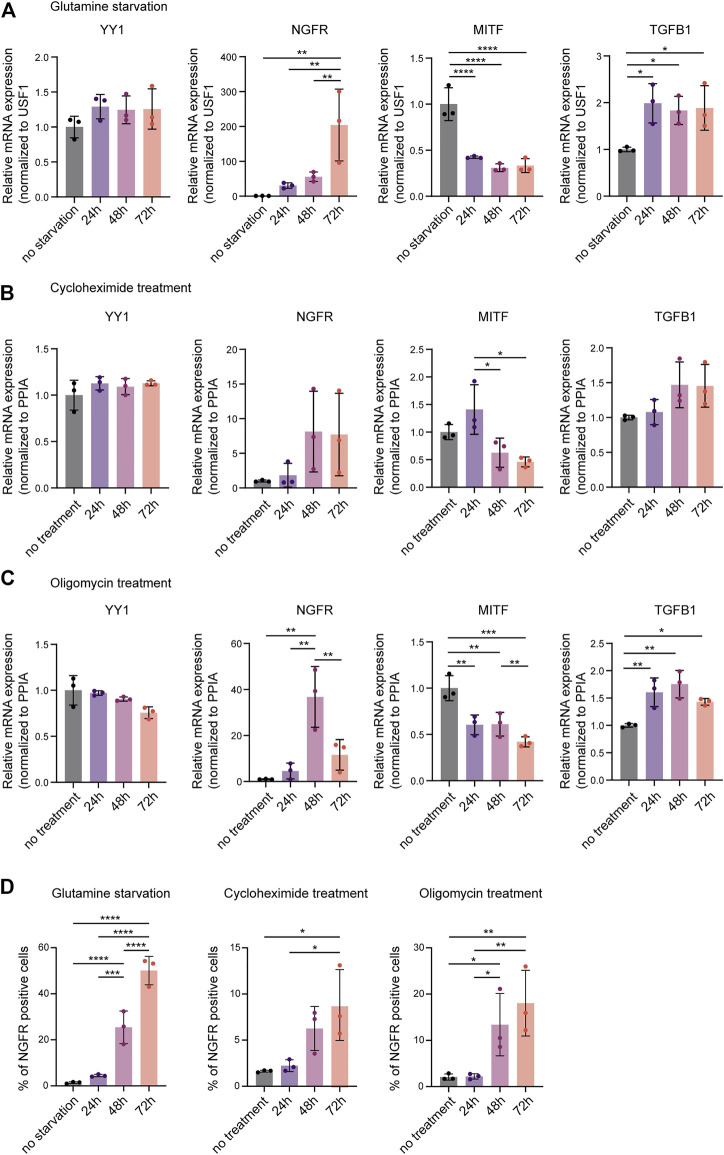
Metabolic stress mimics the invasive gene signature found after YY1 knockdown in melanoma cells. Quantification of mRNA by qRT-PCR for YY1 as well as selected genes involved in EMT and invasion that were found to be altered upon YY1 knockdown in human melanoma cells. Metabolic stress was induced by **(A)** glutamine starvation, **(B)** cycloheximide treatment, **(C)** or oligomycin treatment for 24, 48 and 72 h. Data is shown in fold change relative to control. **(D)** Quantification of NGFR expression by FACS analysis after glutamine starvation, cycloheximide treatment or oligomycin treatment, respectively. All experiments were conducted in independent biological triplicates. *p* value was determined by one-way ANOVA followed by Benjamini, Krieger and Yekutieli for multiple comparison correction with *p*-values: *< 0.05; **<0.01; ***<0.001; ****<0.0001. Not significant is not depicted. Error bars represent mean ± SD.

Re-analysis of published ChIP-seq data did not reveal a direct regulatory effect of YY1 on invasiveness and phenotype switching genes in melanoma (Varum et al., 2019). However, YY1 has been shown to directly bind and regulate a large subset of genes involved in metabolism and protein synthesis (Varum et al., 2019). Its knockdown was found to induce metabolic stress by disturbing numerous cellular metabolic processes leading to metabolic and translational reprogramming in human melanoma cells and interfering with their proliferative capacity (Varum et al., 2019). As metabolic and translational reprogramming can drive melanoma invasion (Falletta et al., 2017) and no evidence for direct binding and suppression of invasiveness genes by YY1 was found, we postulated that metabolic stress caused by YY1 knockdown might mediate siYY1-induced changes in the expression of phenotype switching genes. To support this idea, we exposed human melanoma cells to different metabolic stressors that we have previously found to be associated with loss of YY1 (Varum et al., 2019). Indeed, M010817 cells starved for glutamine upregulated the expression of NGFR and TGFB1 and, at the same time downregulated MITF (Figure 2A). Likewise, interference with oxidative phosphorylation by administration of the drug oligomycin as well as disturbing protein biosynthesis by treating the cells with low doses of cycloheximide led to upregulation of NGFR and TGFB1 accompanied by downregulation of MITF (Figures 2B,C) similar to what has been observed in YY1-knockdown human melanoma cells (Figure 1H). Interestingly, YY1 expression itself was not altered on mRNA levels when cells were starved or drug treated for up to 72 h indicating that YY1 is upstream of these cellular stress responses and is not *per se* affected by metabolic stress (Figures 2A–C). As NGFR is known to be translationally regulated (Huang et al., 2021), we further confirmed changes in NGFR expression on the protein level by FACS analysis and found that all metabolic stress conditions tested resulted in a strong increase in the percentage of NGFR-positive cells over the course of the experiment (Figure 2D). These data demonstrate that, similar to YY1 knockdown itself, interference with YY1-controlled metabolic processes, such as glutamine metabolism, oxidative phosphorylation, and protein biosynthesis, alters the expression of key players in phenotype switching and melanoma invasion. It is therefore conceivable that YY1 knockdown induces an invasiveness gene signature in melanoma cells by activation of a metabolic stress program.

### YY1 Knockdown Sensitizes Melanoma Cells to TGF-β Stimulation and Increases the Expression of Genes Related to TGF-β-Mediated EMT and Invasiveness

Our GSEA of siYY1-treated human melanoma cells revealed members of the TGF-β family, in particular TGFB1, among the most upregulated differentially expressed genes (Figure 1H). Consistently, *Signal Transduction_TGF-beta, GDF and Activin signaling* was one of the top pathways upregulated after YY1 knockdown (Figure 1D). TGF-β signaling is a known inducer of EMT and metastasis in cancer, including melanoma (Hao, Baker and Dijke, 2019; Tuncer et al., 2019; Kim et al., 2020) and has been associated with a cellular stress response and metabolic reprogramming (Jiang et al., 2014; Krstić et al., 2015).

**FIGURE 3:**
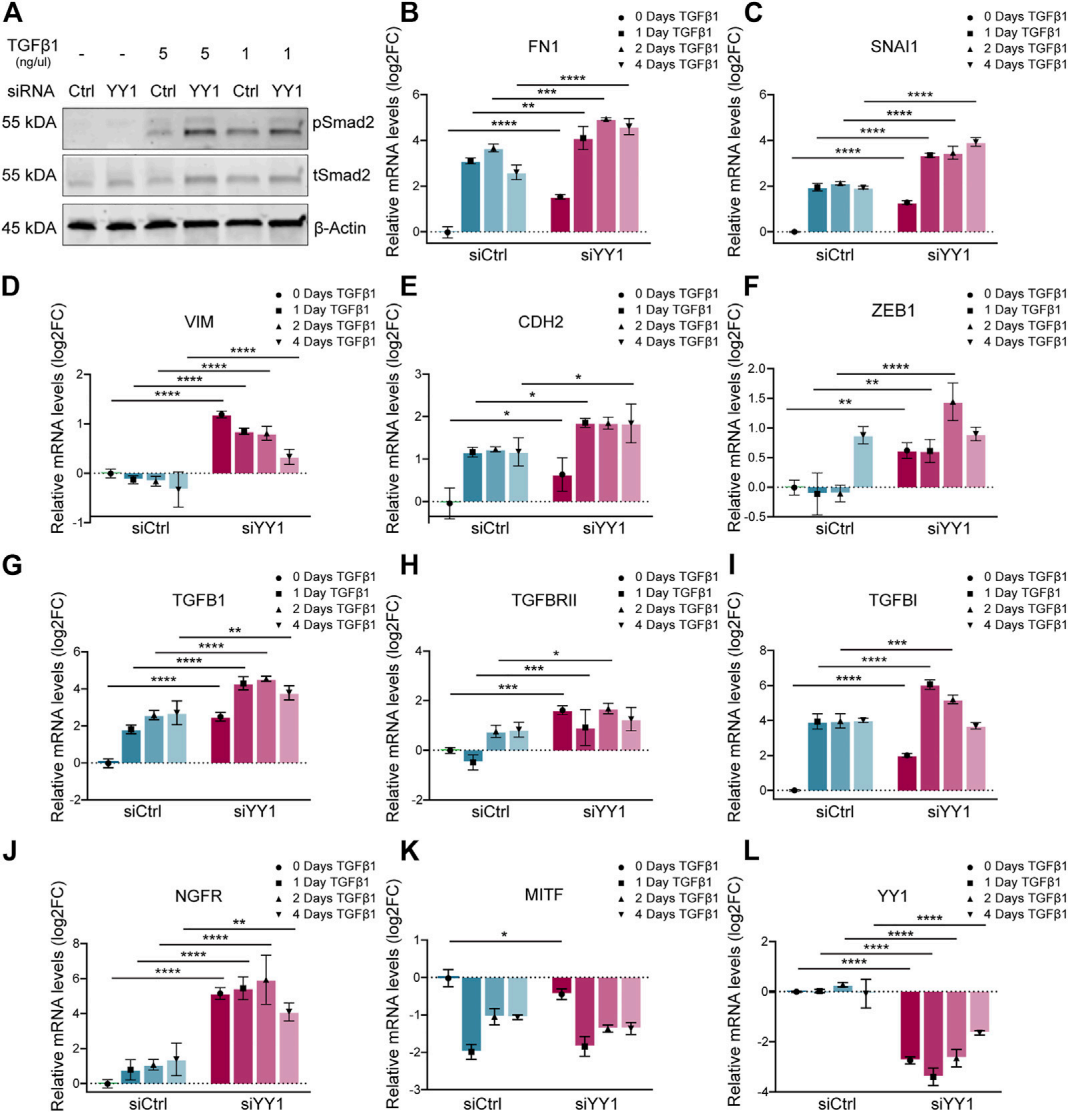
YY1 knockdown in melanoma cell lines increases sensitivity to TGF-β stimulation. **(A)** Western blot to assess TGF-β signaling via phosphorylation of Smad2. Cells were treated with siYY1 and siCtrl for 48 h before stimulation for 30 min with indicated TGF-β1 concentrations. **(B–L)** Quantification of mRNA by qRT-PCR for YY1 as well as for genes involved in EMT and invasion. Cells were either treated with siYY1 or siCtrl and stimulated with 5 ng/µl of TGF-β1 for the specified times. Data is shown in log2 fold change (FC) relative to control. *p* value was determined with two-way ANOVA **(B–L)** with *p*-values: *< 0.05; **<0.01; ***<0.001; ****<0.0001. Not significant is not depicted. Error bars represent mean ± SD.

To find out whether YY1 knockdown influences TGF-β signaling, we analyzed the levels of phosphorylated (p)-Smad2—one of the downstream targets of TGF-β–in YY1-knockdown cells stimulated with TGF-β1. Interestingly, in the presence of TGF-β1, YY1 loss increased Smad2 phosphorylation, indicating that melanoma cells display enhanced sensitivity to TGF-β1 when YY1 levels are low (Figure 3A). TGF-β signaling induces EMT and invasiveness by regulating the expression of several EMT genes (Xu, Lamouille and Derynck, 2009). Intriguingly, YY1 knockdown in combination with TGF-β1 led to increased expression of the EMT genes FN1, SNAI1, VIM, CDH2 and ZEB1 compared to the control group treated with siCtrl and TGF- β1 (Figures 3B–F). TGF-β signaling can function in an autocrine and paracrine manner in melanoma to drive invasiveness and metastasis formation (Daroqui et al., 2012; Tuncer et al., 2019) and TGF-β ligands were described to initiate and regulate their own expression through a positive feedback loop (Perrot, Javelaud and Mauviel, 2013). Consequently, TGF-β1 stimulation increased the expression of TGFB1 and its associated genes TGFBRII and TGFBI, and this effect was even more pronounced upon concomitant knockdown of YY1 (Figures 3G–I). Additionally, a synergistic effect of TGF-β1 treatment and YY1-knockdown was observed for the phenotype switching gene NGFR (Figure 3J). On the opposite, MITF levels were significantly downregulated after TGF-β1 treatment although this effect was not further boosted by siYY1 treatment (Figure 3K). Of note, however, TGF-β1 stimulation did not alter YY1 expression, indicating that YY1 acts upstream of TGF-β (Figure 3L). The combined data suggests that TGF-β signal activation contributes to the enhanced invasiveness program observed upon YY1 knockdown by synergistically boosting the expression of classical EMT genes and influencing other important players in melanoma phenotype switching.

### Knockdown of YY1 in Human Melanoma Cells Leads to Increased Migration and Invasion *In Vitro* and *In Vivo*

**FIGURE 4:**
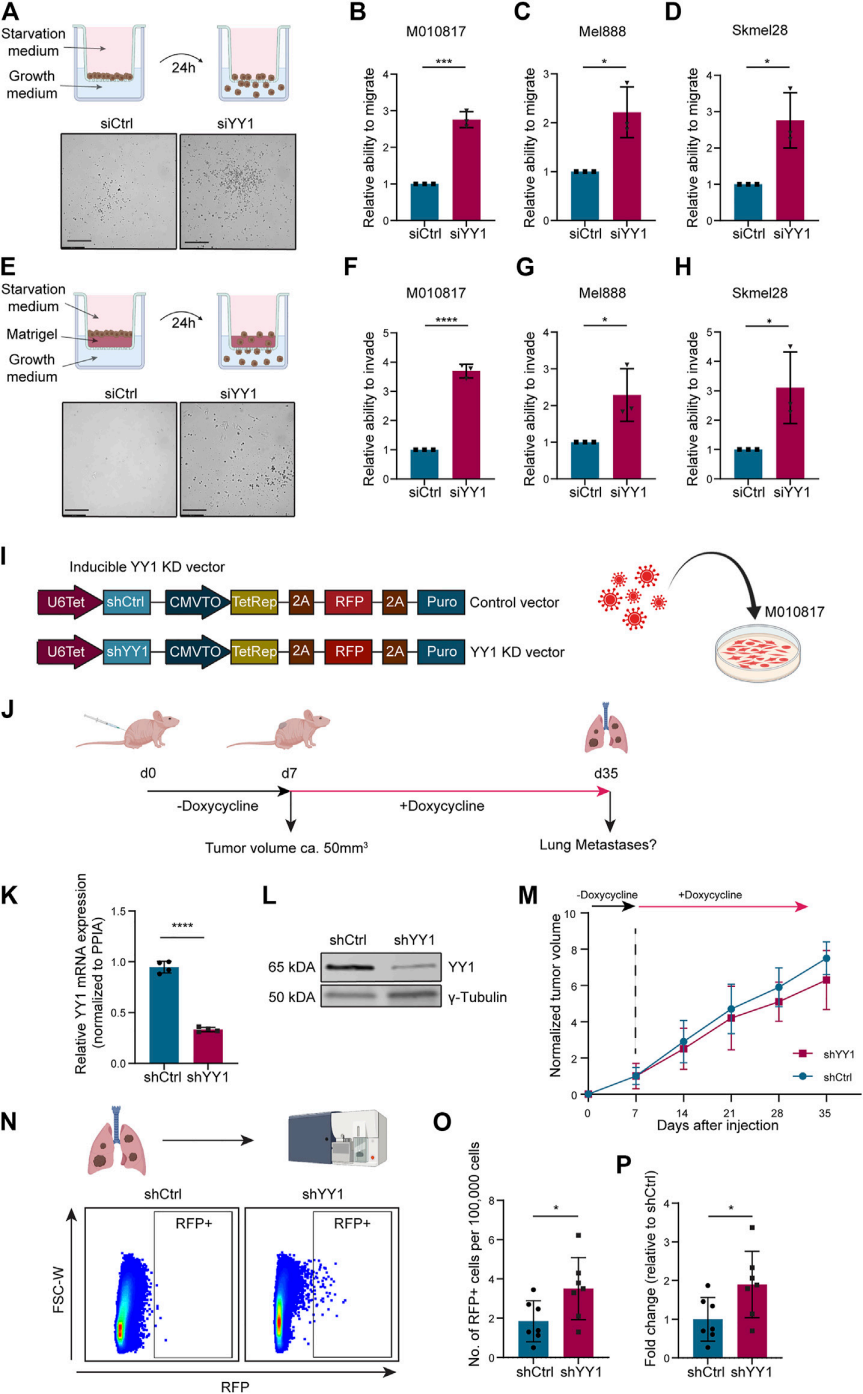
YY1 knockdown leads to increased migration and invasion of human melanoma cells *in vitro* and *in vivo*.. **(A)** Top: Scheme of Corning Transwell ^®^ migration assay. Bottom: Representative pictures of migrated cells after 24 h. Scale bar 250 µm. **(B–D)** Quantification of M010817, Mel888 and Skmel28 cells that migrated through the porous membrane after 24 h. Prior to seeding, cells were treated either with siYY1 or siCtrl for 72 h. Data is shown in fold change relative to control. **(E)** Top: Scheme of Corning Transwell^®^ invasion assay. Bottom: Representative pictures of invading cells after 24 h. Scale bar 250 µm. **(F–H)** Quantification of M010817, Mel888 and Skmel28 cells that invaded through the Matrigel layer and the porous membrane after 24 h. Prior to seeding, cells were treated either with siYY1 or siCtrl for 72 h. **(I)** Left: Scheme of the doxycycline inducible shRNA constructs containing a stably expressed RFP reporter. Right: Scheme of viral transduction. **(J)** Experimental set up of the xenograft *in vivo* metastasis assay. **(K)** Quantification of YY1 mRNA levels *via* qRT-PCR from isolated tumors tissue at experimental end point. **(L)** Western blot confirming YY1 knockdown in tumors at experimental end point. **(M)** Tumor growth curve over the course of the experiment in mm^3^. **(N)** Top panel: Scheme of the FACS analysis and, bottom panel, representative picture of gating strategy to identify RFP-positive cells from the lungs. **(O)** Quantification of RFP + cells in absolute numbers per 100,000 recorded cells. **(P)** Number of RFP + cells presented as fold change relative to control group. *p* value was determined with unpaired Student’s t test with *p*-values: *< 0.05; **<0.01; ***<0.001; ****<0.0001. Error bars represent mean ± SD.

To functionally assess whether YY1 alters the melanoma cell migration and invasion potential, we performed siRNA-mediated YY1 knockdown in three human melanoma cell lines and first subjected them to Corning Transwell ^®^ migration assay chambers (Figure 4A). In all three human melanoma cell lines tested, YY1 knockdown significantly promoted the migration capacity of the cells (Figures 4B–D). Next, to assess the invasiveness of YY1-knockdown cells, siCtrl or siYY1-treated cells were transferred to Corning Transwell^®^ chambers coated with a thin layer of Matrigel (Figure 4E). Again, we observed that siYY1-transfected cells could invade and pass through the Matrigel in higher numbers than siCtrl-transfected cells (Figures 4F–H).

To validate the observed increased invasion potential of human melanoma cells after YY1 knockdown in an *in vivo* model, we generated a human melanoma cell line carrying a tracer (red fluorescent protein, RFP) and a doxycycline-inducible shRNA construct targeting YY1 to be used for grafting into immunocompromised mice (Figure 4I). Next, we injected those cells into the flanks of immunocompromised mice and after tumor establishment at around day 7, treated the animals for 4 weeks with doxycycline in the drinking water to stably knockdown YY1 (Figure 4J). At experimental endpoint, tumors from cells carrying the shYY1 construct showed reduced mRNA and protein levels of YY1 compared to control, confirming the stable knockdown of YY1 upon grafting *in vivo* (Figures 4K,L). Of note, we could not detect a significant difference in tumor growth between YY1 knockdown tumors and control group (Figure 4M) unlike previously reported after complete YY1 depletion (Varum et al., 2019), indicating that the levels of YY1 reduction are relevant for proliferation control of melanoma cells.

To assess the metastatic potential of shYY1 and shCtrl human melanoma cells, we dissected the lungs of the animals at experimental endpoint and performed FACS analysis to isolate RFP-traced melanoma cells from the dissociated tissue (Figure 4N) as described before (Zingg et al., 2018; Diener et al., 2021). Quantification of RFP positive cells within the lungs revealed a significant increase of metastatic cells in animals injected with shYY1 cells compared to control cells (Figures 4O,P).

### Conditional Depletion of YY1 in the Melanocytic Lineage of a Transgenic Murine Melanoma Model Increases Metastasis Formation

**FIGURE 5:**
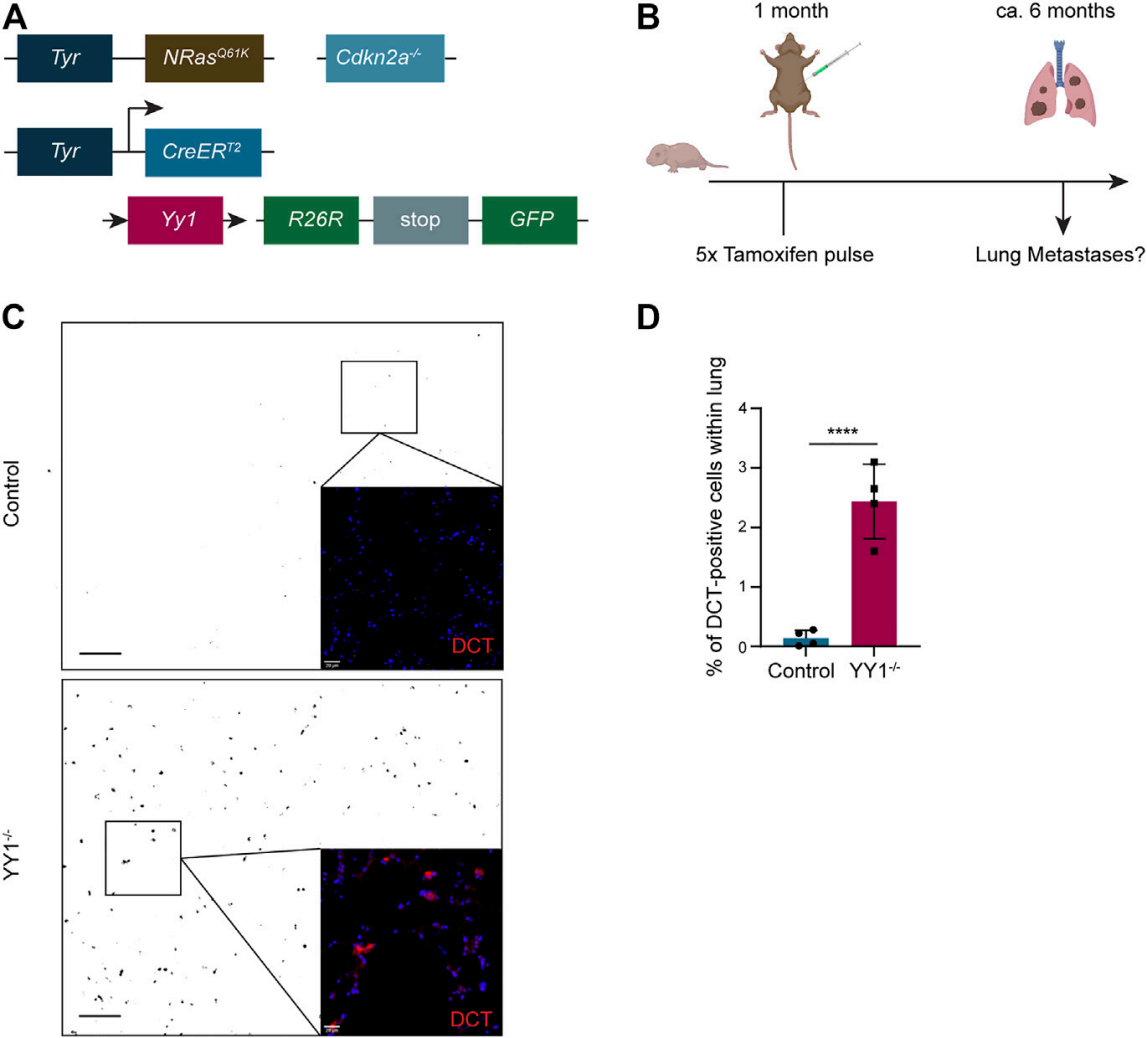
Loss of YY1 in the melanocytic lineage leads to increased metastasis formation in the lung. **(A)** Scheme of the transgenic mouse model. **(B)** Experimental strategy to assess Yy1 loss in tumors and metastasis formation. **(C)** Representative immunohistochemical stainings of lung sections for DCT. Pictures were inverted and set to greyscale except zoomed-in images. Scale bar for greyscale pictures 250 μm; for zoomed-in images 20 µm. **(D)** Quantification of DCT + cells representing lung micrometastases. Number of animals per group *n* = 4. Each dot represents average count of five sections per lung. *p* value was determined with unpaired Student’s t test with *p*-values: *< 0.05; **<0.01; ***<0.001; ****<0.0001. Error bars represent mean ± SD.

*Tyr::N-Ras* ^*Q61K*^ *Cdnk2a* ^*−/−*^ *Tyr::Cre* ^*ERT2*^ *R26R::Stop:EGFP Yy1* ^*lox/lox*^ mice represent a genetically engineered mouse model, in which metastatic melanoma develops spontaneously (Ackermann et al., 2005; Shakhova et al., 2012). Previously, we conditionally ablated *Yy1* in the melanocytic lineage of this model (Figure 5A) to reveal a crucial role of Yy1 in melanoma formation and growth (Varum et al., 2019). To address the question whether Yy1 also plays a role in melanoma cell dissemination and metastasis formation, we analyzed lungs of non-injected *Tyr::N-Ras* ^*Q61K*^ *Cdnk2a* ^*−/−*^ *Tyr::Cre* ^*ERT2*^ *R26R::Stop:EGFP Yy1* ^*lox/lox*^ control mice and tamoxifen-injected *Tyr::N-Ras* ^*Q61K*^ *Cdnk2a* ^*−/−*^ *Tyr::Cre* ^*ERT2*^ *R26R::Stop:EGFP Yy1* ^*lox/lox*^ (*Yy1* knockout) mice for metastatic lesions (Figure 5B). Specifically, we performed immunohistochemical staining for the melanocyte and melanoma-specific marker DCT to identify metastatic lesions (Figure 5C). The validation that the GFP tracer was indeed expressed in cells of the melanocytic lineage and the recombination efficiency after tamoxifen administration were previously reported (Varum et al., 2019). Also, a staining confirming the colocalization of DCT with YY1-recombined GFP-positive cells is shown in the supplemental material (Supplementary Figure S1E). Next, we quantified DCT-positive melanoma cells in both, the lungs of *Yy1* knockout and control animals (Figure 5D). Strikingly, compared to the control, we found a significant increase in DCT-positive cells in tamoxifen-injected *Yy1* knock out animals demonstrating that Yy1 negatively regulates metastatic spreading *in vivo*.

## Discussion

The transcription factor YY1 exhibits numerous roles in tissue homeostasis and development (Verheul et al., 2020). Recently, it has been identified as a master regulator of a NC transcriptional program important for proper NC development (Varum et al., 2019). YY1 also fulfills important functions in cancer, where it can act either as a tumor promoter or tumor suppressor depending on the cancer type (Zhang et al., 2011; Sarvagalla et al., 2019). In the present study we show that, in melanoma, YY1 elicits a dual role: On the one hand, reduced YY1 levels were previously reported to prevent melanoma formation, revealing a role of YY1 in promoting tumor initiation and growth (Varum et al., 2019); on the other hand, we show here that downregulation of YY1 results in increased invasion and micrometastasis formation of melanoma cells *in vitro* and *in vivo*, highlighting a role of YY1 in suppressing melanoma cell invasiveness. Thus, YY1 emerges as a novel regulator of phenotype switching in melanoma, reminiscent of other regulators of NC development that also antagonistically control proliferation vs. invasiveness in melanoma cells (Diener et al., 2021; Diener and Sommer, 2021).

As we did not find YY1 to directly bind and regulate genes involved in invasion, EMT and metastasis formation, it is likely that YY1 exerts its function via indirect regulation of known melanoma invasiveness and EMT genes. To cope with cellular stress–induced by various factors–cancer cells undergo metabolic rewiring affecting pathways, such as glucose and glutamine utilization, mitochondrial and oxidative metabolisms, and reprogramming of protein translation. These metabolic changes are associated with an increased cellular invasion potential (Chiang, Wu and Lee, 2017; García-Jiménez and Goding, 2019). Similarly, cell-intrinsic events, such as oncogene activation or loss of tumor suppressor function, can induce a pseudo-starvation state within melanoma cells leading to a nutrient limitation response in the cell changing the cellular phenotype towards invasion and ultimately driving melanoma metastasis formation (García-Jiménez and Goding, 2019). Since YY1 has been shown to bind and regulate a plethora of genes involved in metabolism and protein biosynthesis and knockdown of YY1 induced metabolic changes in glucose and lipid metabolism, TCA cycle, amino acid and nucleotide metabolism and protein translation (Varum et al., 2019), we hypothesize that YY1 knockdown resembles a state of pseudo-starvation triggering cells to become more invasive. At the same time, metabolic reprogramming by YY1 was shown to interfere with melanoma cell proliferation (Varum et al., 2019) indicating that YY1 exerts its two functions in phenotype switching–promoting proliferation and suppressing invasion–*via* its role as metabolic regulator.

One of the key factors to be downregulated upon YY1 knockdown was MITF–the known master regulator of melanocyte development and differentiation (García-Jiménez and Goding, 2019)—and its target genes DCT, TYRP1 and MLANA, which are involved in pigmentation and pigment cell differentiation (Seberg, Van Otterloo and Cornell, 2017). Goding and colleagues have introduced a rheostat model, according to which MITF expression levels determine the phenotypic state of melanoma cells within the phenotype switching process (Carreira et al., 2006; Tang et al., 2020). Hoek *et al.* (2008) had postulated that low levels of MITF generate a more invasive/less proliferative melanoma cell phenotype (Hoek and Goding, 2010) and, indeed, inhibition of MITF was shown to increase the metastatic potential of melanoma (Cheli et al., 2011), which is in line with our observations. However, YY1 does not directly regulate MITF expression (Varum et al., 2019). Interestingly, translational reprogramming as a cellular response to stress is associated with MITF downregulation (Falletta et al., 2017). Likewise, MITF is downregulated in response to metabolic stress caused by glutamine and glucose shortage (Falletta et al., 2017; Ferguson et al., 2017). Accordingly, in all our metabolic stress experiments, MITF was downregulated, strengthening the hypothesis that phenotype switching induced by loss of YY1 is mediated by metabolic stress affecting, among others, MITF expression levels.

We also observed NGFR to be upregulated both after YY1 knockdown and in our metabolic stress experiments. This is in agreement with previous reports linking NGFR expression to cellular responses to stress, such as therapeutic intervention (Lehraiki et al., 2015), hypoxia (Tong et al., 2018), inflammation (Landsberg et al., 2012) and nutrient starvation (Menon et al., 2014). Since so far, no direct regulatory link between YY1 and NGFR has been reported, the NGFR expression changes observed after YY1 knockdown might be due to metabolic changes induced by YY1 loss. Hence, NGFR upregulation likely reflects a cellular response to metabolic stress, similar to the downregulation of MITF in response to YY1 downregulation. Several studies have shown that NGFR is involved in melanoma cell migration and invasion, establishing it as a mediator of phenotype switching by suppressing melanoma proliferation and simultaneously promoting invasion (Truzzi et al., 2008; Restivo et al., 2017). This was further supported by data from head and neck squamous cell carcinoma (HNSCC) strengthening the link between NGFR expression and increased metastasis (Chung et al., 2018; Lin et al., 2020). Hence, regulation of NGFR by YY1-controlled metabolic programs appears to contribute to increased invasiveness and metastasis formation observed upon reduction of YY1 expression levels in melanoma cells.

In many tumors, upregulation of TGF-β is considered to be pro-oncogenic and pro-metastatic (Massagué, 2008). Also in melanoma, TGF-β signal activation was shown to promote metastasis formation and malignancy in a genetically engineered mouse model (Tuncer et al., 2019). Gene set enrichment analysis of YY1 knockdown melanoma cells revealed *Signal Transduction_TGF-beta, GDF and Activin signaling*, as one of the top upregulated pathways, which encompassed the genes TGFB1, TGFBRI and TGFBRII. This upregulation of TGFB1 after YY1 knockdown is in line with previous reports, where it has been shown that YY1 transcriptionally represses TGFB1 in human renal mesangial cells (Gao et al., 2019). We found that YY1-knockdown melanoma cells were more sensitive to TGF-β1 stimulation, in that YY1 knockdown cells treated with TGF-β1, boosted the expression of the EMT genes *FN1*, *CDH2*, *ZEB1*, *SNAI1* and *VIM*, among others. This synergistic effect of TGF-β1 on YY1 knockdown cells could be explained due to increased expression of the TGF-β1 receptors TGFBR1 and TGFBR2 after YY1 loss and by the repressive function of YY1 on the transcriptional activity of SMADs (Kurisaki et al., 2003), which are crucial intracellular signaling molecules involved in the canonical TGF-β pathway. Of note, TGF-β stimulation has also been reported to induce the expression of NGFR (Restivo et al., 2017) and to repress MITF levels (Nishimura et al., 2010), similarly to what we observed in our YY1 knockdown cells.

In sum, our findings support a model, in which enhanced expression of an invasiveness gene signature in melanoma and, consequently, increased invasiveness and metastasis formation observed upon YY1 knockdown reflects a melanoma cell-specific defense response to metabolic stress–induced by perturbance of multiple YY1-dependent metabolic pathways.

## Data Availability

The original contributions presented in the study are included in the article/Supplementary Material. The RNA-seq datasets of M010718, Mel888 and Skmel28 after siRNA-mediated YY1 knockdown is available at the European Nucleotide Archive (EAN), Accession IDs: PRJEB52505 (for Mel888 and Skmel28) and PRJEB21636 (for M010817).

## References

[B1] Ackermann J. Frutschi M. Kaloulis K. McKee T. Trumpp A. Beermann F. (2005). Metastasizing Melanoma Formation Caused by Expression of Activated N-RasQ61K on an INK4a-Deficient Background. Cancer Res. 65 (10), 4005–4011. 10.1158/0008-5472.CAN-04-2970 15899789

[B2] Carreira S. Goodall J. Denat L. Rodriguez M. Nuciforo P. Hoek K. S. (2006). Mitf Regulation of Dia1 Controls Melanoma Proliferation and Invasiveness. Genes Dev. 20 (24), 3426–3439. 10.1101/GAD.406406 17182868 PMC1698449

[B3] Cheli Y. Giuliano S. Fenouille N. Allegra M. Hofman V. Hofman P. (2012). Hypoxia and MITF Control Metastatic Behaviour in Mouse and Human Melanoma Cells. Oncogene 31 (19), 19312461–19312470. 10.1038/onc.2011.425 21996743

[B4] Chen Q. Zhang J.-J. Ge W.-L. Chen L. Yuan H. Meng L.-D. (2019). YY1 Inhibits the Migration and Invasion of Pancreatic Ductal Adenocarcinoma by Downregulating the FER/STAT3/MMP2 Signaling Pathway. Cancer Lett. 463, 37–49. 10.1016/j.canlet.2019.07.019 31404611

[B5] Chiang T.-S. Wu H.-F. Lee F.-J. S. (2017). ADP-ribosylation Factor-like 4C Binding to Filamin-A Modulates Filopodium Formation and Cell Migration. MBoC 28 (22), 3013–3028. 10.1091/MBC.E17-01-0059 28855378 PMC5662259

[B6] Chung M. K. Jung Y. H. Lee J. K. Cho S. Y. Murillo-Sauca O. Uppaluri R. (2018). CD271 Confers an Invasive and Metastatic Phenotype of Head and Neck Squamous Cell Carcinoma through the Upregulation of Slug. Clin. Cancer Res. 24 (3), 674–683. 10.1158/1078-0432.CCR-17-0866 29208672 PMC6713647

[B7] Daroqui M. C. Vazquez P. De Kier Joffé E. B. Bakin A. V. Puricelli L. I. (2012). TGF-β Autocrine Pathway and MAPK Signaling Promote Cell Invasiveness and *In Vivo* Mammary Adenocarcinoma Tumor Progression. Oncol. Rep. 28 (2), 567–575. 10.3892/OR.2012.1813 22614218 PMC3981025

[B8] Diener J. Baggiolini A. Pernebrink M. Dalcher D. Lerra L. Cheng P. F. (2021). Epigenetic Control of Melanoma Cell Invasiveness by the Stem Cell Factor SALL4. Nat. Commun. 12 (1). 10.1038/S41467-021-25326-8 PMC8379183 34417458

[B9] Diener J. Sommer L. (2021). Reemergence of Neural Crest Stem Cell-like States in Melanoma during Disease Progression and Treatment. STEM CELLS Transl. Med. 10 (4), 522–533. 10.1002/SCTM.20-0351 33258291 PMC7980219

[B10] Falletta P. Sanchez-del-Campo L. Chauhan J. Effern M. Kenyon A. Kershaw C. J. (2017). Translation Reprogramming Is an Evolutionarily Conserved Driver of Phenotypic Plasticity and Therapeutic Resistance in Melanoma. Genes Dev. 31 (1), 18–33. 10.1101/gad.290940.116 28096186 PMC5287109

[B11] Fang Z. Yang H. Chen D. Shi X. Wang Q. Gong C. (2019). YY1 Promotes Colorectal Cancer Proliferation through the miR-526b-3p/E2F1 axis. Am. J. Cancer Res. 9 (12), 2679–2692. 31911854 PMC6943347

[B12] Ferguson J. Smith M. Zudaire I. Wellbrock C. Arozarena I. (2017). Glucose Availability Controls ATF4-Mediated MITF Suppression to Drive Melanoma Cell Growth. Oncotarget 8 (20), 32946–32959. 10.18632/ONCOTARGET.16514 28380427 PMC5464841

[B13] Gao P. Li L. Yang L. Gui D. Zhang J. Han J. (2019). Yin Yang 1 Protein Ameliorates Diabetic Nephropathy Pathology through Transcriptional Repression of TGFβ1. Sci. Transl. Med. 11 (510). 10.1126/SCITRANSLMED.AAW2050/SUPPL_FILE/AAW2050_SM.PDF 31534017

[B14] García-Jiménez C. Goding C. R. (2019). Starvation and Pseudo-starvation as Drivers of Cancer Metastasis through Translation Reprogramming. Cell Metab. 29 (2), 254–267. 10.1016/J.CMET.2018.11.018 30581118 PMC6365217

[B15] Goding C. R. (2011). A Picture of Mitf in Melanoma Immortality. Oncogene 30 (20), 202304–202306. 10.1038/onc.2010.641 21278792

[B16] Gordon S. Akopyan G. Garban H. Bonavida B. (2005). Transcription Factor YY1: Structure, Function, and Therapeutic Implications in Cancer Biology. Oncogene 2525 (8), 81125–81142. 10.1038/sj.onc.1209080 16314846

[B17] Hanahan D. Weinberg R. A. (2011). Hallmarks of Cancer: The Next Generation. Cell 144 (5), 646–674. 10.1016/J.CELL.2011.02.013/ATTACHMENT/3F528E16-8B3C-4D8D-8DE5-43E0C98D8475/MMC1.PDF 21376230

[B18] Hao Y. Baker D. ten Dijke P. (2019). TGF-β-Mediated Epithelial-Mesenchymal Transition and Cancer Metastasis. Ijms 20 (11), 2767. 10.3390/IJMS20112767 PMC6600375 31195692

[B19] Hoek K. S. Eichhoff O. M. Schlegel N. C. Döbbeling U. Kobert N. Schaerer L. (2008). *In Vivo* Switching of Human Melanoma Cells between Proliferative and Invasive States. Cancer Res. 68 (3), 650–656. 10.1158/0008-5472.CAN-07-2491 18245463

[B20] Hoek K. S. Goding C. R. (2010). Cancer Stem Cells versus Phenotype-Switching in Melanoma. Pigment Cell & Melanoma Res. 23 (6), 746–759. 10.1111/J.1755-148X.2010.00757.X 20726948

[B21] Huang F. Gonçalves C. Bartish M. Rémy-Sarrazin J. Issa M. E. Cordeiro B. (2021). Inhibiting the MNK1/2-eIF4E axis Impairs Melanoma Phenotype Switching and Potentiates Antitumor Immune Responses. J. Clin. Investigation 131 (8). 10.1172/JCI140752 PMC8262472 33690225

[B22] Jiang L. Xiao L. Sugiura H. Huang X. Ali A. Kuro-o M. (2014). Metabolic Reprogramming during TGFβ1-Induced Epithelial-To-Mesenchymal Transition. Oncogene 34 (30), 30343908–30343916. 10.1038/onc.2014.321 PMC4387121 25284588

[B23] Khachigian L. M. (2018). The Yin and Yang of YY1 in Tumor Growth and Suppression. Int. J. Cancer 143 (3), 460–465. 10.1002/IJC.31255 29322514

[B24] Kim B. N. Ahn D. H. Kang N. Yeo C. D. Kim Y. K. Lee K. Y. (2020). TGF-β Induced EMT and Stemness Characteristics Are Associated with Epigenetic Regulation in Lung Cancer. Sci. Rep. 10 (1), 1–11. 10.1038/s41598-020-67325-7 32606331 PMC7326979

[B25] Krstić J. Trivanović D. Mojsilović S. Santibanez J. F. (2015). Transforming Growth Factor-Beta and Oxidative Stress Interplay: Implications in Tumorigenesis and Cancer Progression. Oxidative Med. Cell. Longev. 2015, 1–15. 10.1155/2015/654594 PMC4452864 26078812

[B26] Kurisaki K. Kurisaki A. Valcourt U. Terentiev A. A. Pardali K. ten Dijke P. (2003). Nuclear Factor YY1 Inhibits Transforming Growth Factor β- and Bone Morphogenetic Protein-Induced Cell Differentiation. Mol. Cell Biol. 23 (13), 4494–4510. 10.1128/MCB.23.13.4494-4510.2003 12808092 PMC164850

[B27] Landsberg J. Kohlmeyer J. Renn M. Bald T. Rogava M. Cron M. (2012). Melanomas Resist T-Cell Therapy through Inflammation-Induced Reversible Dedifferentiation. Nature 490 (7420), 412–416. 10.1038/nature11538 23051752

[B28] Lehraiki A. Cerezo M. Rouaud F. Abbe P. Allegra M. Kluza J. (2015). Increased CD271 Expression by the NF-kB Pathway Promotes Melanoma Cell Survival and Drives Acquired Resistance to BRAF Inhibitor Vemurafenib. Cell Discov. 1, 15030. 10.1038/CELLDISC.2015.30 27462428 PMC4860830

[B29] Lin C. Ren Z. Yang X. Yang R. Chen Y. Liu Z. (2020). Nerve Growth Factor (NGF)-TrkA axis in Head and Neck Squamous Cell Carcinoma Triggers EMT and Confers Resistance to the EGFR Inhibitor Erlotinib. Cancer Lett. 472, 81–96. 10.1016/J.CANLET.2019.12.015 31838083

[B30] Massagué J. (2008). TGFβ in Cancer. Cell 134 (2), 215–230. 10.1016/J.CELL.2008.07.001 18662538 PMC3512574

[B31] Min H.-Y. Lee H.-Y. (2018). Oncogene-Driven Metabolic Alterations in Cancer. Biomol. Ther. 26 (1), 45–56. 10.4062/BIOMOLTHER.2017.211 PMC5746037 29212306

[B32] Nishimura E. K. Suzuki M. Igras V. Du J. Lonning S. Miyachi Y. (2010). Key Roles for Transforming Growth Factor β in Melanocyte Stem Cell Maintenance. Cell Stem Cell 6 (2), 130–140. 10.1016/J.STEM.2009.12.010 20144786 PMC3437996

[B33] Pavlova N. N. Thompson C. B. (2016). The Emerging Hallmarks of Cancer Metabolism. Cell metab. 23 (1), 27–47. 10.1016/J.CMET.2015.12.006 26771115 PMC4715268

[B34] Perrot C. Y. Javelaud D. Mauviel A. (2013). Insights into the Transforming Growth Factor-β Signaling Pathway in Cutaneous Melanoma. Ann. Dermatol 25 (2), 135. 10.5021/AD.2013.25.2.135 23717002 PMC3662904

[B35] Rambow F. Marine J.-C. Goding C. R. (2019). Melanoma Plasticity and Phenotypic Diversity: Therapeutic Barriers and Opportunities. Genes Dev. 33 (19–20), 1295–1318. 10.1101/GAD.329771.119 31575676 PMC6771388

[B36] Ratnikov B. I. Scott D. A. Osterman A. L. Smith J. W. Ronai Z. A. (2017). Metabolic Rewiring in Melanoma. Oncogene 36 (2), 147–157. 10.1038/ONC.2016.198 27270434 PMC5140782

[B37] Ravindran Menon D. Das S. Krepler C. Vultur A. Rinner B. Schauer S. (2014). A Stress-Induced Early Innate Response Causes Multidrug Tolerance in Melanoma. Oncogene 34 (34), 4448–4459. 10.1038/onc.2014.372 25417704 PMC4442085

[B38] Restivo G. Diener J. Cheng P. F. Kiowski G. Bonalli M. Biedermann T. (2017). The Low Affinity Neurotrophin Receptor CD271 Regulates Phenotype Switching in Melanoma. Nat. Commun. 8 (1). 10.1038/s41467-017-01573-6 PMC5719420 29215016

[B40] Rubinfeld B. Robbins P. El-Gamil M. Albert I. Porfiri E. Polakis P. (1997). Stabilization of β-Catenin by Genetic Defects in Melanoma Cell Lines. Science 275 (5307), 1790–1792. 10.1126/SCIENCE.275.5307.1790 9065403

[B41] Sarvagalla S. Kolapalli S. P. Vallabhapurapu S. (2019). The Two Sides of YY1 in Cancer: A Friend and a Foe. Front. Oncol. 9, 1230. 10.3389/FONC.2019.01230/BIBTEX 31824839 PMC6879672

[B42] Seberg H. E. Van Otterloo E. Cornell R. A. (2017). Beyond MITF : Multiple Transcription Factors Directly Regulate the Cellular Phenotype in Melanocytes and Melanoma. Pigment. Cell Melanoma Res. 30 (5), 454–466. 10.1111/pcmr.12611 28649789 PMC5939569

[B43] Serrano M. Lee H.-W. Chin L. Cordon-Cardo C. Beach D. DePinho R. A. (1996). Role of the INK4a Locus in Tumor Suppression and Cell Mortality. Cell 85 (1), 27–37. 10.1016/S0092-8674(00)81079-X 8620534

[B44] Shakhova O. Zingg D. Schaefer S. M. Hari L. Civenni G. Blunschi J. (2012). Sox10 Promotes the Formation and Maintenance of Giant Congenital Naevi and Melanoma. Nat. Cell Biol. 14 (8), 882–890. 10.1038/ncb2535 22772081

[B45] Tang Y. Durand S. Dalle S. Caramel J. (2020). EMT-inducing Transcription Factors, Drivers of Melanoma Phenotype Switching, and Resistance to Treatment. Cancers 12 (8), 2154. 10.3390/CANCERS12082154 PMC7465730 32759677

[B46] Tirosh I. Izar B. Prakadan S. M. Wadsworth M. H. Treacy D. Trombetta J. J. (2016). Dissecting the Multicellular Ecosystem of Metastatic Melanoma by Single-Cell RNA-Seq. Science 352 (6282), 189–196. 10.1126/SCIENCE.AAD0501 27124452 PMC4944528

[B47] Tong B. Pantazopoulou V. Johansson E. Pietras A. (2018). The P75 Neurotrophin Receptor Enhances HIF-dependent Signaling in Glioma. Exp. Cell Res. 371 (1), 122–129. 10.1016/J.YEXCR.2018.08.002 30092219

[B48] Truzzi F. Marconi A. Lotti R. Dallaglio K. French L. E. Hempstead B. L. (2008). Neurotrophins and Their Receptors Stimulate Melanoma Cell Proliferation and Migration. J. Investigative Dermatology 128 (8), 2031–2040. 10.1038/JID.2008.21 18305571

[B49] Tuncer E. Calçada R. R. Zingg D. Varum S. Cheng P. Freiberger S. N. (2019). SMAD Signaling Promotes Melanoma Metastasis Independently of Phenotype Switching. J. Clin. Investigation 129 (7), 2702–2716. 10.1172/JCI94295 PMC6597210 31039140

[B50] Varum S. Baggiolini A. Zurkirchen L. Atak Z. K. Cantù C. Marzorati E. (2019). Yin Yang 1 Orchestrates a Metabolic Program Required for Both Neural Crest Development and Melanoma Formation. Cell Stem Cell 24 (4), 637–653. e9. 10.1016/j.stem.2019.03.011 30951662

[B51] Verfaillie A. Imrichova H. Atak Z. K. Dewaele M. Rambow F. Hulselmans G. (2015). Decoding the Regulatory Landscape of Melanoma Reveals TEADS as Regulators of the Invasive Cell State. Nat. Commun. 6. 10.1038/NCOMMS7683 PMC4403341 25865119

[B53] Verheul T. C. J. van Hijfte L. Perenthaler E. Barakat T. S. (2020). The Why of YY1: Mechanisms of Transcriptional Regulation by Yin Yang 1. Front. Cell Dev. Biol. 8, 1034. 10.3389/fcell.2020.592164 PMC7554316 33102493

[B54] Wei Q. Qian Y. Yu J. Wong C. C. (2020). Metabolic Rewiring in the Promotion of Cancer Metastasis: Mechanisms and Therapeutic Implications. Oncogene 39 (39), 6139–6156. 10.1038/S41388-020-01432-7 32839493 PMC7515827

[B55] Widmer D. S. Cheng P. F. Eichhoff O. M. Belloni B. C. Zipser M. C. Schlegel N. C. (2012). Systematic Classification of Melanoma Cells by Phenotype-specific Gene Expression Mapping. Pigment Cell & Melanoma Res. 25 (3), 343–353. 10.1111/j.1755-148X.2012.00986.x 22336146

[B56] Wu S. Wang H. Li Y. Xie Y. Huang C. Zhao H. (2018). Transcription Factor YY1 Promotes Cell Proliferation by Directly Activating the Pentose Phosphate Pathway. Cancer Res. 78 (16), 4549–4562. 10.1158/0008-5472.CAN-17-4047/653251/AM/TRANSCRIPTION-FACTOR-YY1-PROMOTES-CELL 29921695

[B57] Xu J. Lamouille S. Derynck R. (2009). TGF-β-induced Epithelial to Mesenchymal Transition. Cell Res. 19 (2), 156–172. 10.1038/CR.2009.5 19153598 PMC4720263

[B58] Zhang Q. Stovall D. B. Inoue K. Sui G. (2011). The Oncogenic Role of Yin Yang 1. Crit. Rev. Oncog. 16 (3–4), 163–197. 10.1615/critrevoncog.v16.i3-4.30 22248053 PMC3386643

[B59] Zingg D. Debbache J. Peña-Hernández R. Antunes A. T. Schaefer S. M. Cheng P. F. (2018). EZH2-Mediated Primary Cilium Deconstruction Drives Metastatic Melanoma Formation. Cancer Cell 34 (1), 69–84. e14. 10.1016/j.ccell.2018.06.001 30008323

[B60] Zingg D. Debbache J. Schaefer S. M. Tuncer E. Frommel S. C. Cheng P. (2015). The Epigenetic Modifier EZH2 Controls Melanoma Growth and Metastasis through Silencing of Distinct Tumour Suppressors. Nat. Commun. 6 (161), 1–17. 10.1038/ncomms7051 25609585

