# Supplementary material for: Loss of YY1, a Regulator of Metabolism in Melanoma, Drives Melanoma Cell Invasiveness and Metastasis Formation
Source: Front Cell Dev Biol. 2022 May 26;10:916033. doi: 10.3389/fcell.2022.916033 (PMC9178194; doi:10.3389/fcell.2022.916033)
Supplement: Supplementary file 1 [file Presentation1.pdf]

Loss of YY1, a regulator of metabolism in melanoma, drives melanoma cell invasiveness and metastasis formation

Ulf Guendisch[1], Benjamin Loos[1], Phil F Cheng[2], Reinhard Dummer[2], Mitchell P Levesque[2], Sandra Varum[1], Lukas Sommer[1]

[1]Institute of Anatomy, University of Zurich, Zurich, Switzerland

[2]Department of Dermatology, University Hospital Zurich, Zurich, Switzerland

SUPPLEMENTARY MATERIAL:

SUPPLEMENTARY FIGURE 1.

SUPPLEMENTARY TABLE 1. Antibodies

SUPPLEMENTARY TABLE 2. Human qRT-PCR primers

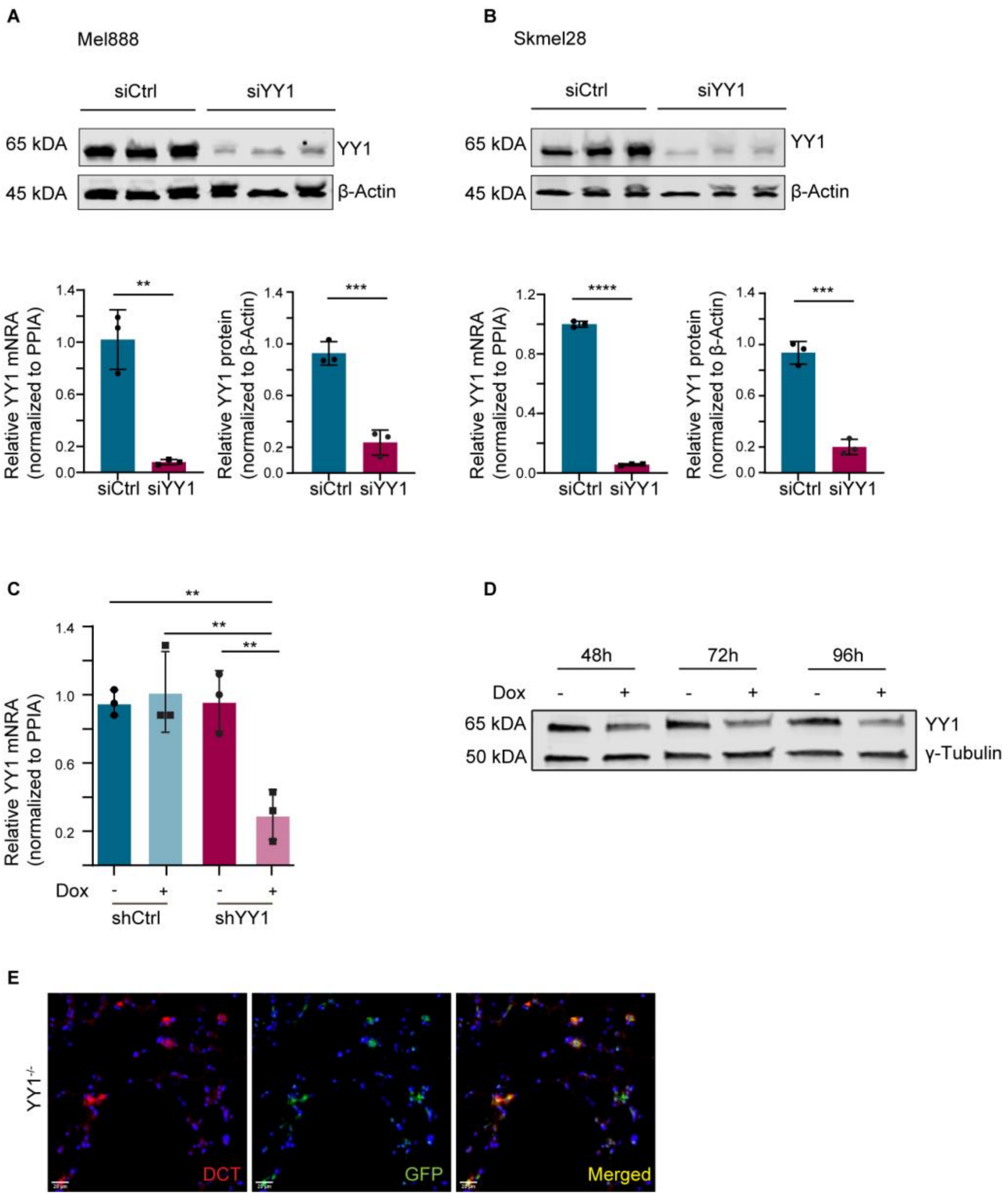

SUPPLEMENTARY FIGURE 1. A+B YY1 expression after 48 hours siRNA-mediated knockdown in Mel888 and Skmel28. Top panel: western blot. Bottom panel: Quantification of qRT-PCR and western blot. All experiments have been performed in independent biological triplicates. Data is shown in fold change (FC) relative to control. **C** Quantification of qRT-PCR results confirming the knockdown efficiency of the shYY1 construct after *in vitro* treatment of M010817 cells with doxycycline for 48 hours. **D** Western blot showing reduced YY1 levels after induction of shYY1 construct with doxycycline for different durations *in vitro.* **E** Representative immunohistochemical stainings of lung sections representing the colocalization of DCT with the melanoma tracer GFP in a YY1$^{-/-}$ animal. Scale bar is set to 20 µm. p value was determined with unpaired Student's t test with p-values: *< 0.05; **<0.01; ***<0.001; ****<0.0001. Error bars represent mean ± SD.

SUPPLEMENTARY TABLE 1. Antibodies

**Antibodies for Stainings**				
**Primary Antibodies**	**Company**	**Cat.Number**	**Host**	**Dilution**
GFP	Aves	GFP-1020	Chicken	1:400
DCT	Pineda	Customized	Rabbit	1:2000
CD271	Alomone Labs	ANT-007	Rabbit	1:200
**Antibodies for Western Blot**				
**Primary Antibody**	**Company**	**Cat.Number**	**Host**	**Dilution**
β-Actin	Sigma Aldrich	A5316	Mouse	1:10000
γ-Tubulin	Sigma Aldrich	T-6557	Mouse	1:10000
YY1 (D5D9Z)	Cell signaling	46395	Rabbit	1:500
pSMAD2 (S465/S467) (E8F3R)	Cell signaling	18338	Rabbit	1:500
Total SMAD2	Abcam	Ab71109	Mouse	1:1000
**Secondary Antibody**	**Company**	**Cat.Number**	**Host**	**Dilution**
IRDye680LT anti-rabbit	Li-Cor	926-68023	Donkey	1:10000
IRDye800CW anti-mouse	Li-Cor	926-32212	Donkey	1:10000
Cy5-conjugated donkey anti-rabbit	Jackson	711-175-152	Donkey	1:300
Cy3-conjugated AffiniPure donkey anti-chicken	Jackson	703-165-155	Donkey	1:300

SUPPLEMENTARY TABLE 2. Human qRT-PCR primers

**Target**	**Forward**	**Reverse**
β-Actin	GGACTTCGAGCAAGAGATGG	AGGAAGGAAGGCTGGAAGAG
PPIA	TTCATCTGCACTGCCAAGAC	TCGAGTTGTCCACAGTCAGC
USF1	CTGCTGTTGTTACTACCCAGG	TCTGACTTCGGGGAATAAGGG
NGFR	CCTACGGCTACTACCAGG ATG	CACACGGTGTTCTGCTTGT
FN1	CAGTGGGAGACCTCGAGAAG	TCCCTCGGAACATCAGAAAC
CDH2	ACAGTGGCCACCTACAAAGG	CCGAGATGGGGTTGATAATG
MITF	GCCTCCAAGCCTCCGATAAG	CATCTGCTCACGCATGAGTTG
YY1	CCTCTCAGATCCCAAACAACT	GCCTTTATGAGGGCAAGCTAT
SNAI1	CCTCCCTGTCAGATGAGGAC	CCAGGCTGAGGTATTCCTTG
VIM	GAGAACTTTGCCGTTGAAGC	GCTTCCTGTAGGTGGCAATC
TGFBI	CACTCTCAAACCTTTACGAGACC	CGTTGCTAGGGGCGAAGATG
TGFB1	CAATTCCTGGCGATACCTCAG	GCACAACTCCGGTGACATCAA
ZEB1	GCACAACCAAGTGCAGAAGA	CATTTGCAGATTGAGGCTGA
TGFBRII	ACGTGTTGAGAGATCGAGG	CCCAGCACTCAGTCAACGTC
